# P-1754. An Antimicrobial Stewardship Program (ASP) Without Antibiotic Restrictions - Performance Update

**DOI:** 10.1093/ofid/ofae631.1917

**Published:** 2025-01-29

**Authors:** Kimberly D Leuthner

**Affiliations:** University Medical Center of Southern Nevada, Las Vegas, Nevada

## Abstract

**Background:**

In 2011, University Medical Center of Southern Nevada (UMC) in Las Vegas presented introductory results of a simple, yet effective ASP program with the unique strategy of not restricting antibiotics. Since this time, ASP has continued to strive towards appropriate antimicrobial use in an attempt to improve patient care and minimize development of resistance. This project is an update of the progress of this ASP program in terms of resistance, antibiotic use and costs.

**Figure:**
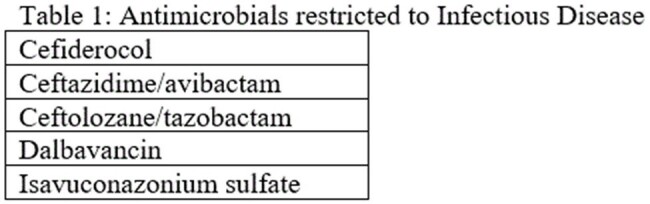

**Methods:**

ASP started January 2008 with minimal resources. Baseline activities included targeted medication use evaluation (MUE), duration regulation, expansion of infection prevention and education. In contrast to many ASP programs our program did not restrict antibiotics – formulary agents were available for any practitioner. Over time activities were adjusted, reporting technologies advanced although personnel resources remained limited. ASP modifications:

- Added MUE agents
- Tiered sensitivity reporting
- Reduce duration to 7 days
- Expand service to 6 days per week

Deviating from initial procedures, in 2015 we implemented restriction of 5 “last-line” antimicrobials to UMC employed Infectious Disease Physicians (table 1) using the EHR. Markers of ASP progress was monitored by rates of organism isolation per 1000 patient days and antimicrobial in days of therapy per 1000 patient days. Financial implications were based on antibiotic expenditures per adult patient day.

**Figure:**
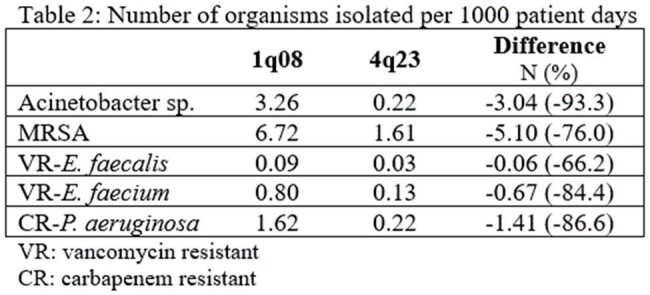

**Results:**

Monitored bacterial pathogens decreased 65 – 94% (table 2). The largest reduction was with Acinetobacter sp. followed by carbapenem resistant *P. aeruginosa*. Antibiotic use also improved (table 3). Overall use was decreased 71.3 ± 14.7% with the largest changes with fluoroquinolones and piperacillin/tazobactam. Of note, not all antibiotics reported are MUE, and use of these agents still improved. Despite expanding formulary, and cost monitoring activities, antibiotic expenditures decreased 47% per adult patient day.

**Figure:**
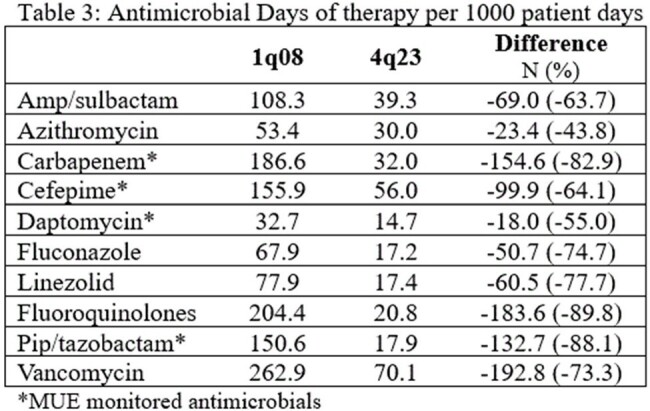

**Conclusion:**

ASP at UMC has demonstrated that simple programs, with limited resources, can decrease the use of unnecessary antimicrobial agents and isolation of specific bacterial pathogens. Additional benefits include a decrease in hospital costs without requiring significant restriction of antimicrobial agents.

**Disclosures:**

**Kimberly D. Leuthner, PharmD, FIDSA**, AbbVie Inc.: Advisor/Consultant|AbbVie Inc.: Honoraria|Basilea Pharamceutica: Advisor/Consultant|La Jolla Pharmaceutical Company: Honoraria|Merck: Advisor/Consultant|Merck: Honoraria|Shionogi, Inc: Honoraria

